# CoAP_UAD: CoAP under attack dataset — A comprehensive dataset for CoAP-based IoT security research

**DOI:** 10.1016/j.dib.2025.112210

**Published:** 2025-10-21

**Authors:** Jose Aveleira-Mata, Álvaro Michelena, Isaías García-Rodríguez, José Luis Calvo-Rolle, Carmen Benavides, Esteban Jove

**Affiliations:** a University of León, Department of Electric, Systems and Automatics Engineering, León, Spain; b University of A Coruña, CTC, CITIC, Department of Industrial Engineering, A Coruña, Spain

**Keywords:** IoT security, Constrained application protocol (CoAP), Protocol-level attacks, Intrusion detection systems (IDS), Anomaly detection, Cyberattack dataset, IoT traffic analysis

## Abstract

Internet of Things (IoT) systems increasingly rely on lightweight protocols such as the Constrained Application Protocol (CoAP), which are designed for resource-constrained devices but open new avenues for protocol-level attacks. The heterogeneity of deployments and the prevalence of UDP-based communication make CoAP networks susceptible to threats that exploit protocol semantics rather than implementation bugs. In this context, studying effective detection methods helps preserve the reliability and safety of constrained IoT deployments.

In this work we introduce CoAP_UAD (CoAP Under Attack Dataset), a publicly documented dataset created to study and benchmark intrusion detection for CoAP-based IoT networks. The attacks in CoAP_UAD deliberately target CoAP protocol behaviors rather than implementation- or device-specific vulnerabilities, abstracting away system particulars. The dataset was produced in a realistic testbed that emulates constrained devices and typical CoAP deployments while executing a diverse suite of protocol-oriented attacks, including three protocol-oriented attack families such as cross-protocol interaction over UDP (e.g., DNS traffic injected into CoAP exchanges), message-format and negotiation manipulation, Denegation of Service (Block size amplification attack). Network traffic was captured at the router, and each frame/flow was labeled and exported to a consistent, CSV format to enable reproducible experimentation.

Specifications Table

**Table:** 

Subject	Computer Sciences
Specific subject area	IoT security with the CoAP protocol: labelled network traffic for detecting protocol-level attacks (cross-protocol over UDP, Man-in-the-Middle, and Denegation of Service) in a realistic CoAP testbed.
Type of data	Three tables in CSV format (68 features per frame)
Data collection	A realistic CoAP IoT testbed was deployed (Node.js CoAP server with observe, ESP8266+DHT11 client, auxiliary clients, and an attacker host). All LAN traffic was captured at the OpenWRT router and converted from PCAP to labelled CSV with a fixed field schema (frame-level + all CoAP fields + type). Three attack scenarios were executed targeting protocol behaviors: cross-protocol over UDP (DNS into CoAP flows), MitM (Ettercap filter over UDP/CoAP), and Denial of Service (DoS) via block size amplification. The resulting files are: CoAP_Cross_protocol.csv (62,943 frames: 60,453 normal; 2490 attack), CoAP_MitM.csv (24,684 frames: 21,222 normal; 3462 attack), and CoAP_DoS.csv (30,319 frames: 21,269 normal; 9050 attack).
Data source location	University of León (RIASC / SECOMUCI), León, Spain; in collaboration with University of A Coruña (CTC, CITIC, Department of Industrial Engineering), A Coruña, Spain.
Data accessibility	Repository name: Figshare. Data identification number: 10.6084/m9.figshare.26362876 Direct URL to data: https://figshare.com/articles/dataset/CoAP_UAD_CoAP_Under_Attack_Dataset_A_public_dataset_for_the_detection_of_attacks_in_IoT_networks_using_CoAP_protocol/26362876
Related research article	

## 1 Value of the Data

• Protocol-level, implementation. Attacks over CoAP behaviors (cross-protocol over UDP, message/negotiation manipulation, blockwise-amplification DoS) rather than device- or vendor-specific flaws.
• Realistic constrained-IoT setup. Router-level captures in a working CoAP testbed reflect UDP loss/reordering and typical traffic patterns.
• Consistent, transparent schema. Three CSVs share a fixed 68-feature per-frame layout, facilitating quick baselines and feature interpretability.
• Reproducible comparisons. Fixed scenarios and standardized labels (Normal, MitM, DoS, Cross-protocol) support benchmarking and repeatable reporting.

## 2 Background

The Constrained Application Protocol (CoAP) is a lightweight application-layer protocol that usually runs over UDP. It follows a REST style (GET/POST/PUT/DELETE) and supports the Observe extension for efficient push/subscribe on constrained devices, which is common in sensors and real-time monitoring [1]. Because DTLS is not always enabled in deployments, model and anomaly-based intrusion detection can play an important role alongside transport security.

Model-and anomaly-based Intrusion Detection Systems (IDS) monitor network traffic to learn a baseline of normal behavior and flag deviations, without modifying endpoints or protocols, as passive sensors, they can be added to existing IoT infrastructures with no changes to devices or applications [2]. A survey reports that deep-learning anomaly detectors align better with heterogeneous, evolving IoT environments than signature-based systems [3], practical deployments are feasible under resource limits via machine learning improved detection on common IoT benchmarks [4].

In IoT there are some CoAP-focused datasets, but the options are relatively limited compared to other protocols. Two notable efforts are CoAP-IoT [5], which uses a simulated environment with message-modification anomalies, and CoAP-DoS [6], centered on denial-of-service scenarios, separate study employing the dataset presented here under CoAP DoS scenarios validates its utility [7] . This leaves room for protocol-level datasets that are built from realistic setups and align with the CoAP specification.

We created the COAP_UAD dataset [8] by building a working CoAP testbed comprising a Node.js server (node-coap), a physical DHT11 sensor on a NodeMCU (ESP-CoAP), a JavaScript client on a Raspberry Pi using Observe for live updates, and Copper (Copper4Cr) clients. Traffic was captured on an OpenWRT router and converted to CSV, retaining frame-level fields, all CoAP fields, and a label column (type).

## 3 Data Description

CoAP_UAD is composed of three CSV files, each corresponding to a specific attack scenario. The detailed characteristics are:

• CoAP_DoS.csv: 30,319 frames in total, with 9050 attack frames (DoS) and 21,269 normal frames.
• CoAP_MitM.csv: 24,684 frames in total, with 3462 attack frames (MitM) and 21,222 normal frames.
• CoAP_Cross_protocol.csv: 62,943 frames in total, with 2490 attack frames (C-P) and 60,453 normal frames.

All CSV files in CoAP_UAD share the same set of 85 fields derived from the Wireshark Display Filter Reference [9] and follow a common schema. The fields are grouped as follows:

• **Common (frame-level) fields**: 28 Fields present in every frame, regardless of whether CoAP is used. Because non-CoAP traffic may appear during attacks, including these fields helps IDS models capture general network patterns beyond IoT device exchanges. A detailed list and descriptions are provided in Table 1.

**Table 1:** Description of common fields.

Field Name	Description	Type
frame.time_delta	Delta time from the previously captured frame	Time offset
frame.time_delta_displayed	Delta time from the previously displayed frame	Time offset
frame.time_invalid	Label a time as invalid	Label
frame.time_relative	Time elapsed since the first frame	Time offset
ip.src	Source IP address	IPv4 Address
ip.dst	Destination IP address	IPv4 Address
tcp.srcport	Source port	Unsigned integer, 2 bytes
tcp.dstport	Destination port	Unsigned integer, 2 bytes
eth.src	Source MAC address	Ethernet or other MAC address
eth.dst	Destination MAC address	Ethernet or other MAC address
frame.cap_len	Length of the captured frame	Unsigned integer, 4 bytes
frame.coloring_rule.name	Coloring rule for the name	String
frame.coloring_rule.string	Coloring rule for the string	String
frame.comment	Frame comments	String
frame.comment.expert	Expert comments	Label
frame.encap_type	Encapsulation type	Signed integer, 2 bytes
frame.file_off	File offset	Signed integer, 8 bytes
frame.ignored	If the frame is ignored	Boolean
frame.incomplete	If the frame is incomplete	Label
frame.interface_id	Interface identifier	Unsigned integer, 4 bytes
frame.interface_name	Interface name	String
frame.len	Wired frame length	Unsigned integer, 4 bytes
frame.link_nr	Link number	Unsigned integer, 2 bytes
• **Common fields**: 56 Fields specific to the CoAP protocol (e.g., version, type, code, message ID, options such as Observe and Block-wise). These describe only frames that use CoAP. A detailed list and descriptions are provided in Table 2.

**Table 2:** Description of CoAP fields.

Field name	Description	Type
coap.block	Block	Frame number
coap.block.count	Block count	Unsigned integer (32 bits)
coap.block.error	Block defragmentation error	Frame number
coap.block.multiple_tails	Block has multiple tails	Boolean
coap.block.overlap	Block overlap	Boolean
coap.block.overlap.conflicts	Block overlapping with conflicting data	Boolean
coap.block.reassembled.in	Reassembled in	Frame number
coap.block.reassembled.length	Reassembled block length	Unsigned integer (32 bits)
coap.block.too_long	Block too long	Boolean
coap.block_length	Block Length	Unsigned integer (32 bits)
coap.block_payload	Block Payload	Byte sequence
coap.blocks	Blocks	Label
coap.code	Code	Unsigned integer (8 bits)
coap.invalid_option_number	Invalid Option Number	Label
coap.invalid_option_range	Invalid Option Range	Label
coap.length	Length	Unsigned integer (32 bits)
coap.mid	Message ID	Unsigned integer (16 bits)
coap.ocount	Opt Count	Unsigned integer (8 bits)
coap.opt.accept	Accept	Character string
coap.opt.block_mflag	More Flag	Unsigned integer (8 bits)
coap.opt.block_number	Block Number	Unsigned integer (32 bits)
coap.opt.block_size	Encoded Block Size	Unsigned integer (8 bits)
coap.opt.ctype	Content-type	Character string
coap.opt.delta	Opt Delta	Unsigned integer (8 bits)
coap.opt.delta_ext	Opt Delta extended	Unsigned integer (16 bits)
coap.opt.desc	Opt Desc	Character string
coap.opt.end_marker	End of options marker	Unsigned integer (8 bits)
coap.opt.etag	Etag	Byte sequence
coap.opt.hop_limit	Hop Limit	Unsigned integer (8 bits)
coap.opt.if_match	If-Match	Byte sequence
coap.opt.if_none_match	If-None-Match	Byte sequence
coap.opt.jump	Opt Jump	Unsigned integer (8 bits)
coap.opt.length	Opt Length	Unsigned integer (8 bits)
coap.opt.length_ext	Opt Length extended	Unsigned integer (16 bits)
coap.opt.location	Location	Character string
coap.opt.location_path	Location-Path	Character string
coap.opt.location_query	Location-Query	Character string
coap.opt.max_age	Max-age	Unsigned integer (32 bits)
coap.opt.name	Opt Name	Character string
coap.opt.object_security_expand	Expanded Flag Byte	Boolean
coap.opt.object_security_kid	Key ID	Byte sequence
coap.opt.object_security_kid_context	Key ID Context	Byte sequence
coap.opt.object_security_kid_context_len	Key ID Context Length	Unsigned integer (8 bits)
coap.opt.object_security_kid_context_present	Key ID Context Present	Boolean
coap.opt.object_security_kid_present	Key ID Present	Boolean
coap.opt.object_security_non_compressed	Non-compressed COSE message	Boolean
coap.opt.object_security_piv	Partial IV	Byte sequence
coap.opt.object_security_piv_len	Partial IV Length	Unsigned integer (8 bits)
coap.opt.object_security_reserved	Reserved	Boolean
coap.opt.object_security_signature	Signature Present	Boolean
coap.opt.observe	Observe	Unsigned integer (32 bits)
coap.opt.opt_echo	Echo	Byte sequence
coap.opt.opt_no_response	No-Response	Unsigned integer (8 bits)
coap.opt.opt_ocf_accept_version	OCF-Accept-Content-Format-Version	Unsigned integer (8 bits)
coap.opt.opt_ocf_version	OCF-Content-Format-Version	Unsigned integer (8 bits)
coap.opt.opt_request_tag	Request-Tag	Byte sequence
coap.opt.payload_desc	Payload Desc	Character string
coap.opt.proxy_scheme	Proxy-Scheme	Character string
coap.opt.proxy_uri	Proxy-Uri	Character string
coap.opt.size1	Size1	Unsigned integer (32 bits)
coap.opt.subscr_lifetime	Lifetime	Unsigned integer (32 bits)
coap.opt.token	Token	Character string
coap.opt.unknown	Unknown	Byte sequence
coap.opt.uri_auth	Uri-Authority	Character string
coap.opt.uri_host	Uri-Host	Character string
coap.opt.uri_path	Uri-Path	Character string
coap.opt.uri_path_recon	Uri-Path	Character string
coap.opt.uri_port	Uri-Port	Unsigned integer (16 bits)
coap.opt.uri_query	Uri-Query	Character string
coap.optcount	Option Count	Unsigned integer (8 bits)
coap.option_length_bad	Option length bad	Label
coap.option_object_security_bad	Invalid Object-Security Option Format	Label
coap.option_oscore_bad	Invalid OSCORE Option Format	Label
coap.oscore_kid	OSCORE Key ID	Byte sequence
coap.oscore_kid_context	OSCORE Key ID Context	Byte sequence
coap.oscore_piv	OSCORE Partial IV	Byte sequence
coap.payload	Payload	Character string
coap.payload_desc	Payload Desc	Character string
coap.payload_length	Payload Length	Unsigned integer (32 bits)
coap.request_first_in	Retransmission of request in	Frame number
coap.response_first_in	Retransmission of response in	Frame number
coap.response_in	Response In	Frame number
coap.response_time	Response Time	Time offset
coap.response_to	Request In	Frame number
coap.retransmitted	Retransmitted	Label
coap.tid	Transaction ID	Unsigned integer (16 bits)
coap.token	Token	Byte sequence
coap.token_len	Token Length	Unsigned integer (8 bits)
coap.type	Type	Unsigned integer (8 bits)
coap.unknown_option_number	Unknown Option Number	Label
coap.version	Version	Unsigned integer (8 bits)
• **Frame labelling:** A special field, type, labels each frame by class: “DoS” (denial of service), “MitM” (man-in-the-middle), “C-P” (cross-protocol), or “Normal”. This field is summarized in Table 3.

**Table 3:** Tagging of frames based on their state.

Field name	Description	Type
Type	Tag frames as normal or under attack	``normal'' / ``DoS'' / ``MitM'' / ``intrusion''

## 4 Experimental Design, Materials and Methods

### 4.1 CoAP environment

We set up a real testbed that generates routine CoAP activity and attack traffic, with non-CoAP background traffic to reflect a mixed environment. The setup comprises an embedded temperature–humidity sensor node, a Node.js CoAP server with a REST-style API and observe support, and a lightweight JavaScript client running on a Raspberry Pi 3 that subscribes to the observe resource to receive continuous updates. All packet-level interactions are captured in .pcap format for subsequent analysis and labeling. The following subsections describe the access network, the CoAP server, the IoT sensor device, and the client applications.

• **The network:** All devices are attached to the same local area network, which enables controlled experiments and full-packet capture using standard tools (e.g., tcpdump/tshark). This configuration ensures that every CoAP/UDP exchange is stored for subsequent inspection and dataset curation.
• **CoAP server:** Implemented in Node.js using the node-coap library [10], the server exposes REST-style services using JSON payloads. A POST to /temp receives temperature and humidity readings, processes them, and stores them in a Node.js-based database. A GET to /temp returns the latest stored temperature. The server also integrates observe at the /obs resource, continuously streaming temperature and humidity updates to subscribed clients.
• **IoT device (temperature sensor):** A NodeMCU board is connected to a DHT11 temperature–humidity sensor (. The board is programmed with ESP-CoAP to send readings via CoAP at regular intervals [11], producing a steady benign flow of traffic toward the server.
• **JavaScript client:** A lightweight client application written in JavaScript uses node-coap to connect to the server, subscribe to the observe resource, and print each update to the terminal. This client runs on a Raspberry Pi 3, as the workload is modest and does not require significant resources.
• **Copper4Cr clients:** Two clients based on Copper4Cr are employed [12]Copper4Cr is a free and open-source CoAP client (the successor of the original “Copper” add-on) that provides a graphical interface to issue CoAP requests, discover resources, and use observe, which is useful for interactive testing and debugging.

**Fig. 1:**
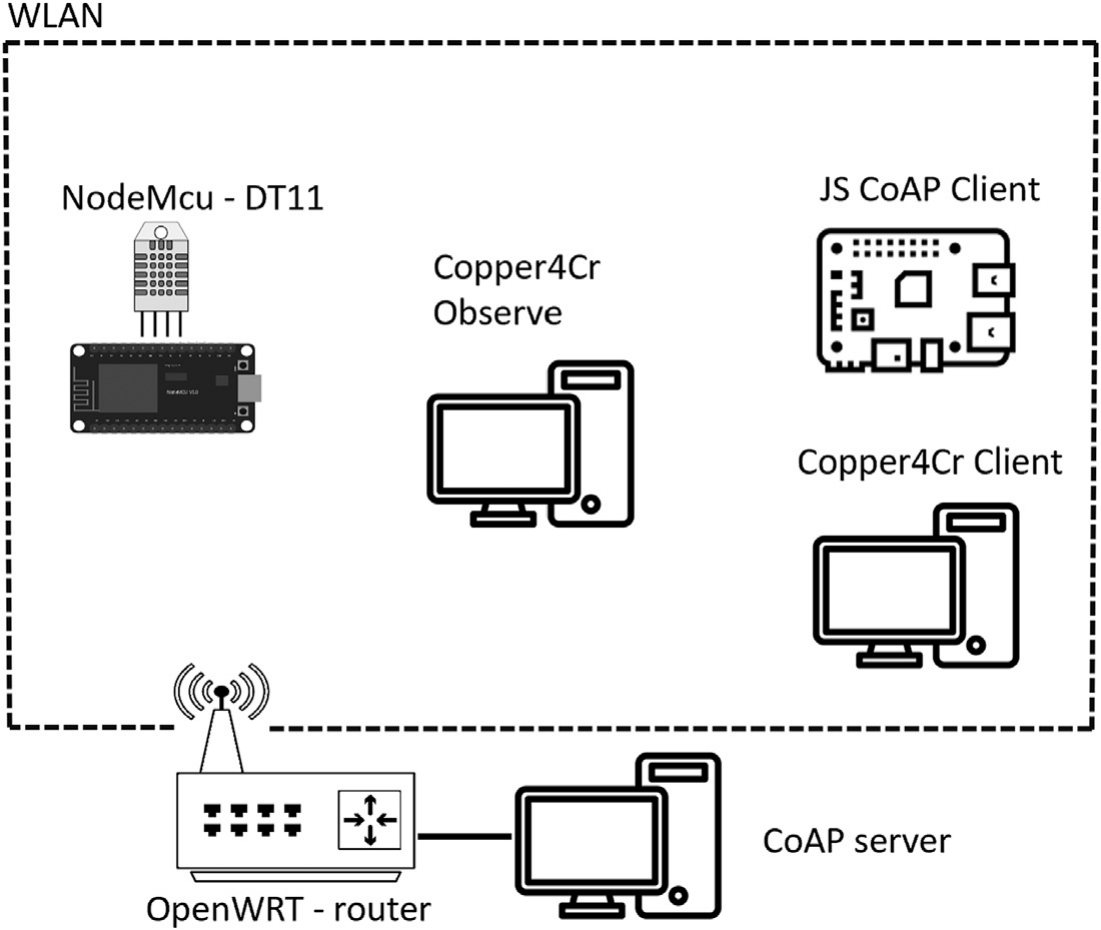
CoAP environment.

A CoAP testbed (Fig. 1) was developed to create a dataset for anomaly detection. In accordance with RFC 7252 [13], protocol-level threats were reproduced in three areas: (1) amplification risk (Sec. 11.3, “Risk of Amplification”); (2) attacks arising from parsing and URI handling, such as man-in-the-middle (Sec. 11.1, “Parsing the Protocol and Processing URIs”); and (3) cross-protocol attacks leveraging other UDP-based protocols (Sec. 11.5, “Cross-Protocol Attacks”).

### 4.2 Amplification-based DoS

CoAP servers often return responses that are larger than the requests, especially when the Block-wise Transfer option is used. Because CoAP can negotiate very small block sizes, an attacker can force the server to split a resource into many response blocks. This property can make CoAP clients vulnerable to denial-of-service (DoS).

Within the experimental environment, a server answers a CoAP client (Copper4Cr) while an attacker runs on a Linux virtual machine. The attack relies on IP spoofing: the attacker forges the client’s IP address so that the server directs its responses to the victim client. To maximize traffic toward the victim, the attacker negotiates very small block sizes, causing the server to send a large number of response packets.

On the attacker machine, iptables is used as follows: iptables -t nat -A POSTROUTING -p udp -o eth0 -j SNAT –to IPVICTIM:PORT

**Fig. 2:**
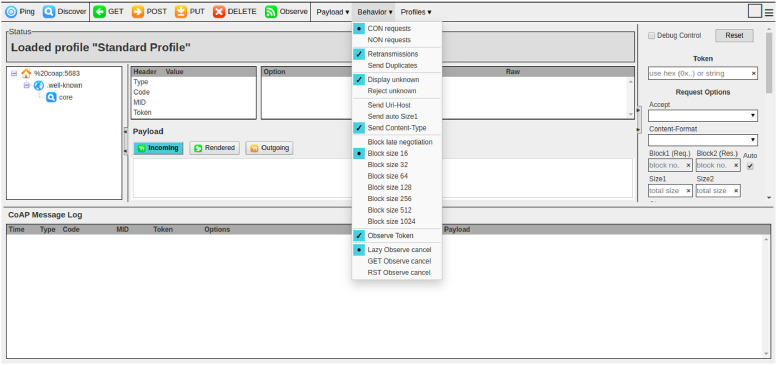
Changing the Block Size in Copper4Cr.

Here, IPVICTIM is the victim client’s address and PORT is the UDP port used by the CoAP exchange. The attacker then runs Copper4Cr as the victim does and selects Block size 16 (the smallest block). Requests are issued by the attacker; no replies reach the attacker, while the victim client receives a high volume of unsolicited response packets (Fig. 2).

It is also possible to script the negotiation of the smallest CoAP block size using a Node.js client. In CoAP, the Block2 option (number 23) controls the response block size; setting szx = 0 requests 16-byte blocks.

### 4.3 Man-in-the-middle attack

The attack begins by observing the client–server exchange (“sniffing”) and then altering selected messages to trigger an error. A CoAP client and server communicate normally, while the attacker host (Kali Linux) is placed between them using a standard interception technique so that traffic passes through the attacker’s machine. Ettercap is employed to intercept and modify CoAP messages in transit.

Ettercap filters are used by compiling source rules into binaries that the program can apply on the fly. In this case, an existing HTTP/TCP filter was adapted to CoAP/UDP, leveraging the similar idea of a request path. Concretely, whenever the client issues a GET or POST to “/temp,” the filter replaces that path with “/mitm” on the attacker host. As a result, the server receives a request to a non-existent resource and does not answer the client’s original request. From the client’s perspective, the operation appears normal, because the alteration happens in transit and the client believes it sent the correct path.

### 4.4 DNS-to-CoAP cross-protocol

This attack sends a Domain Name System (DNS) packet from the attacker to the client while spoofing the server’s IP address. The client, which is running a CoAP Observe request, interprets the incoming packets as if they were sent by the CoAP protocol. CoAP is exposed to this vector because of its similarity to other UDP-based protocols, such as DNS. DNS itself maps human-readable domain names to IP addresses so networked devices can communicate. This technique can evade firewall rules tailored to CoAP and can be used to mount denial-of-service attacks or even inject code into different clients in the IoT system [6].

**Fig. 3:**
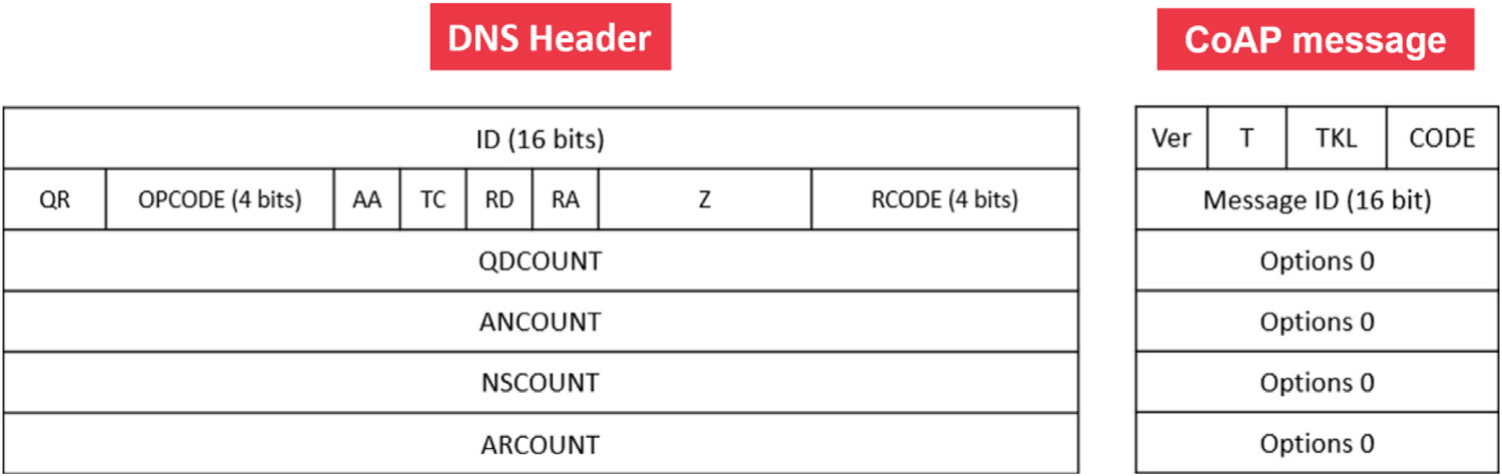
Comparison between the DNS header and a CoAP message.

In CoAP, the ID field is a 16-bit identifier used to match requests and responses. The T field is a 2-bit integer indicating the message type. TKL is a 4-bit integer for the Token length, and CODE is an 8-bit integer indicating the request/response type. There is also a Ver field, a 2-bit integer that is always 1 (binary 01) and indicates the CoAP version. Because of CoAP’s simple frame structure, an attacker can align the 16 bits of the ID field with the 16 bits formed by T, TKL, CODE, and Ver, creating forged CoAP packets. To perform these attacks, a Node.js script can be used on the attacker host with the native-dns-packet library [14]This library allows editing each parameter of a DNS packet, making it possible to adjust field sizes and contents so they match the bit lengths and port associated with a CoAP message Fig. 3.

## Limitations

Not applicable.

## Ethics Statement

The authors have read and follow the ethical requirements for publication in Data in Brief. This work does not involve human subjects, animal experiments, or data collected from social media platforms.

The dataset was generated in a controlled laboratory testbed and contains only technical network traffic and CoAP protocol fields. No personally identifiable information (PII) is included.

## Credit Author Statement

Jose Aveleira-Mata: created the dataset, verified the labels, and wrote the manuscript. Álvaro Michelena: validation, Writing- Reviewing and Editing. Isaías García-Rodríguez: Data curation, Visualization, Validation, Writing- Reviewing and Editing. José Luis Calvo-Rolle: Conceptualization, Supervision, Writing- Reviewing and Editing. Esteban Jove: supervision, Writing- Reviewing and Editing. Carmen Benavides: provided overall guidance during conceptualization and dataset creation and reviewed the manuscript.

## Data Availability

figshareCoAP_UAD (Original data)
